# Pressure Anisotropy in Polymer Brushes and Its Effects on Wetting

**DOI:** 10.1021/acs.langmuir.3c03727

**Published:** 2024-02-15

**Authors:** Lars B. Veldscholte, Jacco H. Snoeijer, Wouter K. den Otter, Sissi de Beer

**Affiliations:** † Functional Polymer Surfaces, Department of Molecules and Materials, MESA+ Institute, University of Twente, 7500 AE Enschede, The Netherlands; ‡ Physics of Fluids, MESA+ Institute, University of Twente, 7500 AE Enschede, The Netherlands; § Multiscale Mechanics, Department of Fluid and Thermal Engineering, MESA+ Institute, University of Twente, 7500 AE Enschede, The Netherlands

## Abstract

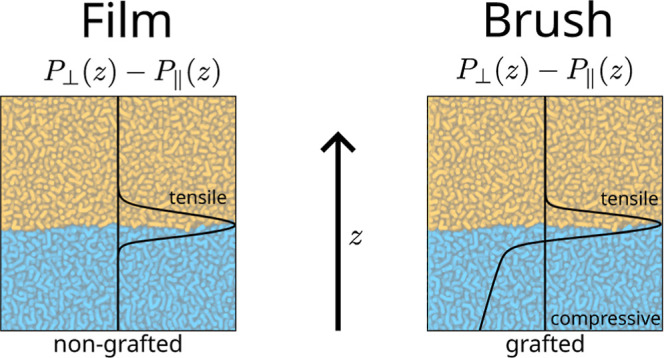

Polymer brushes, coatings consisting of densely grafted macromolecules, experience an intrinsic lateral compressive pressure, originating from chain elasticity and excluded volume interactions. This lateral pressure complicates a proper definition of the interface and, thereby, the determination and interpretation of the interfacial tension and its relation to the wetting behavior of brushes. Here, we study the link among grafting-induced compressive lateral pressure in polymer brushes, interfacial tension, and brush wettability using coarse-grained molecular dynamics simulations. We focus on grafting densities and polymer–liquid affinities such that the polymer and liquid do not tend to mix. For these systems, a central result is that the liquid contact angle is independent of the grafting density, which implies that the grafting-induced lateral compressive pressure in the brush does not influence its wettability. Although the definition of brush interfacial tensions is complicated by the grafting-induced pressure, the difference in the interfacial tension between wet and dry brushes is perfectly well-defined. We confirm explicitly from Young’s law that this difference offers an accurate description of the brush wettability. We then explore a method to isolate the grafting-induced contribution to the lateral pressure, assuming the interfacial tension is independent of grafting density. This scenario indeed allows disentanglement of interfacial and grafting effects for a broad range of parameters, except close to the mixing point. We separately discuss the latter case in light of autophobic dewetting.

## Introduction

Polymer brushes are coatings consisting of macromolecules that are end-grafted to a substrate at sufficiently high grafting densities so that they are forced to stretch away. They then form a so-called “brush” structure, with the polymer chains extending upward from the substrate. These systems show considerably different behaviors compared to bulk polymers or nongrafted films. Coatings consisting of polymer brushes have several applications in stabilization of colloids,^1^ sensors,^2,3^ separation membranes,^4−7^ low-friction^8,9^ and antifouling coatings,^10^ and stimulus-responsive materials^11^ such as smart adhesives.^12^ In several of these applications, polymer brushes are applied in air,^13^ as opposed to immersed in liquid. In these cases, understanding of wetting behavior of polymer brushes is imperative, especially since brushes are known to display counterintuitive wetting effects.^14−16^

**Figure 1:**
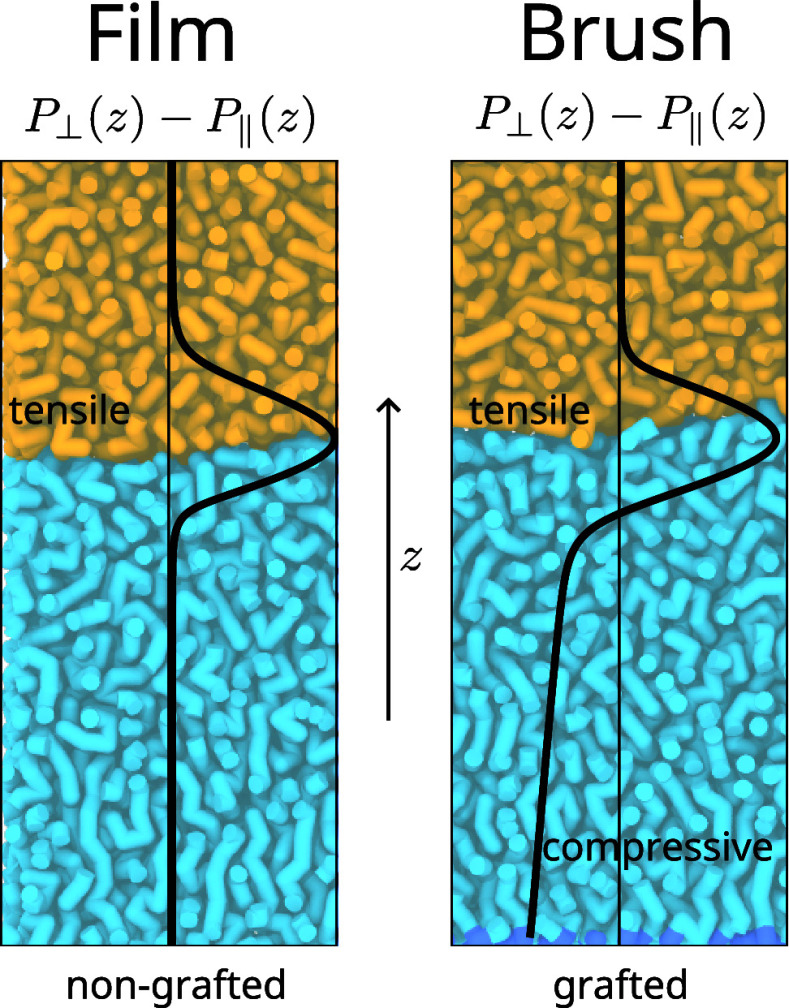
Illustrations of pressure anisotropy profiles (left) for a liquid on top of a nongrafted film, and (right) for a liquid on top of a brush. In both cases, a tensile lateral pressure localized near the interface, whose integral is naturally associated with the surface tension, arises. The pressure anisotropy profile for a liquid on top of a brush shows an additional grafting-induced compressive pressure within the brush.

A polymer brush’s conformation is the result of a competition between the polymer chains’ entropic elasticity, which tends to contract the brush, and excluded volume interactions between the chains, which are repulsive and thereby extend the brush. This competition also produces an inherent lateral compressive pressure in polymer brushes: the pressure inside the brush is anisotropic with an effective compression parallel to the grafting plane. As sketched in Figure 1, this pressure is largest at the grafting plane and smoothly reaches zero at the brush surface, as shown by both self-consistent field theory^17^ and molecular dynamics (MD) simulations.^18,19^ This grafting-induced lateral pressure can even bend a flexible substrate.^17,20^ No such lateral pressure exists in nongrafted polymer films.

**Figure 2:**
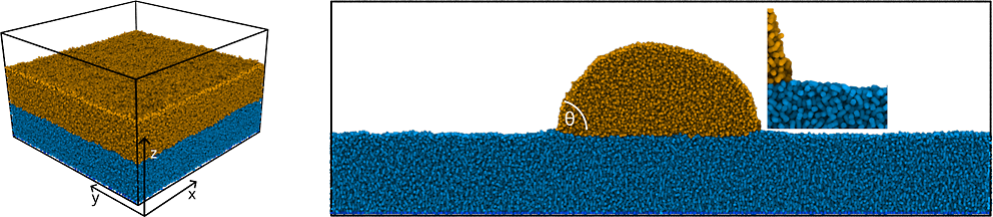
Snapshots of the two types of simulation setups. (Left) A liquid layer on top of a brush or film in a box with a square ground plane. (Right) Cross-section of a cylindrical droplet on a brush or film in a rectangular quasi-2D setup. The inset zooms in on the contact line, highlighting the absence of a wetting ridge. The smooth wall supporting the brush or film from below is not shown.

Polymer brushes are able to adsorb and absorb solvents, just like nongrafted polymer films. Whereas the latter will dissolve in a good solvent, the grafted polymers of a brush can merely swell, forming a diffuse polymer–solvent interfacial region in the process. An interesting situation arises when the solvent conditions are less favorable such that a sharp interface will form. An interfacial tension will emerge, which manifests itself as a lateral tensile pressure localized near the interface, as illustrated in Figure 1. To make this more explicit, we define the pressure anisotropy as
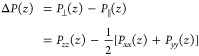
1where *P*_*xx*_, *P*_*yy*_, and *P*_*zz*_ denote the three diagonal elements of the Cartesian pressure tensor **P**, with *P*_⊥_ and *P*_∥_ the pressures perpendicular and parallel, respectively, to the flat substrate. The definitions of the Cartesian directions are given in Figure 2, in a 3D snapshot of a MD simulation. In this setup, the planar symmetry of the system means that the pressure parallel to the interface is a function of *z* only, *P*_∥_(*z*) = *P*_*xx*_(*z*) = *P*_*yy*_(*z*), while mechanical equilibrium dictates a constant normal pressure equal to the isotropic bulk pressure in the fluid, *P*_⊥_ = *P*_*zz*_ = *P*_fluid_.^21,22^ While it is difficult to measure the pressure distribution in a brush experimentally, it can be extracted from MD simulations^23,24^ with relative ease. The interfacial tension is then obtained as the integral of Δ*P*(*z*) over the interface^18,21,25,26^
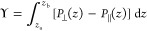
2where the integration boundaries *z*_a_ and *z*_b_ are located in the bulk phases on either side of the interface.

The above equation for the interfacial tension offers a clear physical interpretation for the interface between two immiscible liquids, where the pressure anisotropy is nonzero only in a small region near the interface. The same applies to the interface between a polymer melt and a liquid. For a brush–liquid interface, however, it is less clear what exactly constitutes the interfacial region. This can be seen from the sketched pressure anisotropy profiles in Figure 1: the pressure anisotropy is negative (compressive) in the brush bulk, and grows more negative with increasing distance from the interface.^18^ Therefore, it is not straightforward to determine the actual “interfacial excess” that defines the thermodynamic property of the interface. Instead, we are left with an unpleasant situation where the value of the interfacial tension varies with the choice of the integration boundary in the brush, *z*_a_.

The compressive pressure anisotropy within the brush is due to grafting and not due to interfacial interactions, and hence, it would be inappropriate to treat the full integral as a traditional liquid–liquid interfacial tension. Including (part of) the brush bulk can even yield a negative interfacial tension if the grafting-induced compressive pressure is high enough compared to the peak in the interfacial region. This result led to various interpretations.

For example, Dimitrov et al.^18^ include the entire brush in the interfacial tension calculation and conclude that this interfacial tension is fundamentally different from a conventional liquid–liquid interfacial tension: it is not limited to positive values, and a change in sign is not accompanied by a phase transition. Moreover, they propose that the pressure anisotropy profile can be separated in a “brush” and an “interfacial” part by splitting at the height *z*_0_ where the anisotropy vanishes, Δ*P*(*z*_0_) = 0. Badr et al.^27^ “choose to evaluate only the integral over the peaks at the interfaces”. Similarly, Milchev and Petkov^28^ noted negative interfacial tensions that grow in absolute value with increasing grafting density in concave polymer brushes. Léonforte and Müller^16^ extracted interfacial tensions from the thermal fluctuation spectrum of the brush–vapor interface, reporting good agreement with the surface tensions of their nongrafted counterparts. Whereas integrating only a selected part of the pressure anisotropy curve yields promising results, it is not evident whether this approach includes all interfacial contributions or whether it includes interfacial contributions only.

In this paper, we will explore the link between the negative pressure anisotropy in brushes and the interfacial tensions and how this affects the wettability of the brush. We will use coarse-grained MD simulations, focusing on the two types of configurations, illustrated in Figure 2: a layer of liquid deposited on top of the brush, resulting in a planar interface, and an infinitely long cylindrical droplet on top of the brush. The former simulation yields interfacial tensions, and the latter probes the wettability by means of the contact angle of the droplet. Finally, we will compare the results against similar configurations of nongrafted polymer films to explore whether the pressure anisotropy due to grafting and due to the presence of the interface can be disentangled.

## Surface Tensions, Surface Energies, and Wetting

In spite of difficulties associated with defining interfacial tensions of brushes, their observable wetting properties should be well defined and are only a function of the difference between the brush–liquid and brush–vapor interfacial energies. This follows from Young’s law, which describes the contact angle of a liquid droplet (*L*) resting on a solid substrate (*S*) in equilibrium with its vapor (*V*):
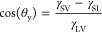
3where the γ denotes interfacial free energies per unit area and the two subscripts specify the interface.

Two possible complications may arise here. First, Young’s law applies to rigid surfaces, which polymer brushes and polymer films are not. The brushes simulated here, however, are sufficiently stiff that Young’s law can be used to estimate contact angles.^26^ Second, for elastic substrates the interfacial free energy density γ is not necessarily equal to the interfacial tension Υ as defined in eq 2. The two quantities are related by d(γ*A*) = Υ d*A*, where *A* denotes the interfacial area. For simple liquids, these quantities always take on the same value, i.e., γ = Υ, and one does not explicitly distinguish between the two concepts. In general, however, a local stretching of the interface can change the interfacial composition and thus alter its free energy density. For elastic substrates, this phenomenon is known as the Shuttleworth effect,^26,29−32^ while a similar distinction between interfacial tension and energy arises for surfactants.^33^ When stretching a brush laterally, a constant number of grafting points *N*_g_ becomes distributed over a larger area, and consequently, the grafting density ρ_g_ = *N*_g_/*A* decreases. The change in interfacial free energy is evaluated as
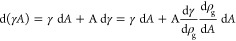
4and it follows that the interfacial tension Υ is related to the interfacial free energy density γ by
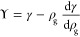
5

By integrating the pressure anisotropy as outlined in eq 2, one obtains the interfacial tension, which for brushes does not necessarily equal the interfacial energy.

Previous simulations by Léonforte et al.^34^ of droplets on brushes of intermediate grafting densities show the contact angle to be independent of the grafting density. Experimental results of contact angles on brushes of varying grafting density also indicate no change in wettability above a certain value.^35,36^ These observations suggest that dγ/dρ_g_ must either vanish or at least be identical for bush–liquid (SL) and brush–vapor (SV) interfaces. Under this convenient condition, one finds
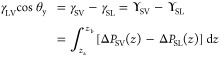
6

This direct relation between contact angles and pressure–anisotropy profiles is explored below.

## Model and Methods

### Model

MD simulations were performed using the LAMMPS^37^ MD software. Coarse-grained MD allows for simulating the relatively large time and length scales of the physical behavior of interest. We use the chemically aspecific Kremer–Grest model,^38^ which has been successfully applied to qualitatively reproduce generic physical behavior of polymers.^8,39−42^ We therefore expect the results reported here to be qualitatively representative of dense brushes of flexible polymer chains wetted by poor solvents. In this model, polymers are represented as freely jointed bead–spring chains. The nonbonded interaction between particles *i* and *j* is described by the Lennard–Jones potential,
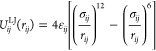
7where *r*_*ij*_ is the interparticle distance, ε_*ij*_ represents the depth of the energy well and all particle pairs share a zero-crossing distance σ_*ij*_ = σ. The potential is truncated at *r*_c_ = 2.5 σ,
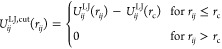
8

Bonded interactions between consecutive beads along a polymer chain are described by the purely attractive finitely extensible nonlinear elastic (FENE) potential
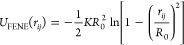
9combined with the purely repulsive Weeks–Chandler–Anderson (WCA) potential
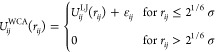
10

The spring constant *K* = 30 ε/σ^2^ and the maximum bond length *R*_0_ = 1.5 σ of the FENE potential are taken from the Kremer–Grest model.^38^ Throughout this work, we will use reduced Lennard–Jones units^25^ with ε and σ serving as the unit of energy and length, respectively. Time is expressed in units of , where *m* is the particle mass, and temperatures are given in ε/*k*_B_, with *k*_B_ the Boltzmann constant.

In addition to the polymer, a liquid consisting of Kremer–Grest tetramers is introduced into the system. We use tetramers instead of monomers to depress the vapor pressure, thereby reducing the vapor density to virtually zero. Hence, the vapor looks empty in the snapshots of the system presented here. The liquid beads are of the same size and mass as the polymer beads and also interact with each other and with the polymer by a Lennard–Jones potential. The liquid (*l*) and polymer (*p*) self-interaction energies are set at ε_ll_ = ε_pp_ = 1.5ε. They are given identical values for simplicity; the value of 1.5 ε is chosen to make the polymer sufficiently stiff to suppress the formation of a wetting ridge at the contact line (see the inset to Figure 2). To control the affinity between polymer and solvent, and thereby the wettability, the polymer–solvent interaction energy ε_pl_ is varied between 0.5ε and 1.5ε, resulting in contact angles of 0–130°. Note that these Lennard–Jones interactions effectively capture all kinds of interactions between polymer beads, rather than merely the van der Waals interaction between two atoms, so combining rules are not applicable here and the ε_*ij*_ parameters can be varied independently of each other.^44^

A system consisting of a rectangular box with periodic boundary conditions along *x* and *y* is set up. Two different box sizes are used: a 3D system of Cartesian dimensions 100 σ × 100 σ × 60 σ for the slab simulations, and a quasi-2D geometry of 250 σ × 10 σ × 80 σ for the droplet simulations. In the latter system, the droplet is periodically continued in the *y* direction to create a cylindrical droplet and thereby eliminate line tension contributions to the contact angle, while the periodic repeat distance is still sufficiently short to suppress Plateau–Rayleigh instabilities.^16,45,46^Figure 2 shows snapshots of both simulation setups, rendered using Ovito.^47^ The brushes and polymer films rest on a flat structureless mathematical wall (*w*) exerting a Lennard–Jones 9–3 potential with ε_pw_ = 1ε and σ_pw_ = 1σ, where the zero-crossing height defines *z* = 0. At the top of the box, a repulsive harmonic potential with spring constant of 100 ε/σ^2^ prevents evaporated fluid molecules from escaping. A monodisperse polymer brush is created by “grafting” polymer chains of *N* = 50 beads to immobile grafting beads positioned randomly in the *z* = 0 bottom plane of the box. This chain length is chosen as a balance between computational cost and adequate reproduction of the polymer brush behavior. The density of grafting points varies between 0.2 and 0.6 σ^–2^, which ensures that all our systems are in the brush regime. Specifically, brushes will form “holes” or crystallize for densities below or above this range, respectively.

### Simulation Procedure

Data files comprising initial configurations of fully stretched Kremer–Grest polymer brushes in rectangular boxes at various grafting densities are generated using a Python script, made available online.^48^

The systems were equilibrated by a short energy minimization using the conjugate gradient method (min_style cg), followed by a dynamics run for 250 τ with a displacement limit (fix nve/limit) of 1 σ per time step. The equilibration was performed with a Langevin thermostat (fix langevin) with a temperature of 1.7 ε/*k*_B_ and a damping parameter of 10 τ. This was followed by a longer dynamics run for 5000 τ without the limit and a less viscous Langevin thermostat (damping parameter of 100 τ) that cooled the system to a temperature of 0.85ε/*k*_B_. This procedure was chosen to equilibrate the polymer brush system as efficiently as possible. In these equilibration runs, a time step of 0.005 τ was used.

For the production runs, the rRESPA multitimescale integrator^49^ was used with an outer time step of 0.010 τ and a 2-fold smaller inner time step of 0.005 τ. This resulted in nonbonded pair interactions being computed every 0.010 τ, but bonded interactions being computed twice as often. Equilibrium simulations sampled the canonical ensemble by thermostatting to a temperature of 0.85ε/*k*_B_ using a Nosé–Hoover chain (fix nvt) with a damping parameter of 0.1 τ. Production runs of the brushes were run for 10^4^ τ, storing density and pressure profiles over *z* at regular intervals. Subsequently, either slabs or cylindrical droplets of liquid were deposited on top of the brushes. The simulations with slabs were run for 10^4^ τ, storing density and pressure profiles over *z*, while droplets were simulated for 10^5^ τ, storing 2D density profiles over *x* and *z*. A similar procedure was applied for the polymer films; the number of polymer chains in these films corresponded to a grafting density of ρ_g_ = 0.4 σ^–2^. To ensure only data from systems in equilibrium were included in the analyses, the first half of each production run was discarded and only the latter half was analyzed.

### Data Analysis

Computing the pressure anisotropy profile Δ*P*(*z*) necessitates a definition of the local pressure tensor, which is not uniquely defined for inhomogeneous systems.^21,22,24,50−52^ The kinetic pressure contribution by particle *i* is readily assigned to the bin along the *z* direction holding the particle. For the virial contribution by the interaction between the *i*–*j* particle pair, we use the Irving–Kirkwood contour: the contribution is distributed over all bins from *i* to *j* in proportion to the fraction of the total distance from *i* to *j* traversed in each of these bins.^23,24^ This distribution of the virial over the bins has the appealing feature of *P*_⊥_(*z*) being constant, in agreement with mechanical equilibrium along the *z* direction.^22,24^ We note that the integrals of the pressure profiles *P*_⊥_(*z*) and *P*_∥_(*z*) over the entire height of the simulation box are independent of the chosen distribution. Galteland et al.^53^ implemented this algorithm in LAMMPS (compute stress/Cartesian) for particles interacting by nonbonded pair forces. We amended the routine to include the bond forces in our polymers; this code is merged in LAMMPS and available as of the 15 June 15, 2023 release.

Contact angles are extracted from the 2D density profiles of droplets by circle fitting, in the spirit of refs (27, 45, 54, 55). Data processing, analysis, and visualization were performed using Python, with NumPy,^56^ SciPy,^57^ and Matplotlib.^58^ The Python code, e.g., to parse LAMMPS ave/chunk output or to extract contact angles from droplets, is made available online.^59^

## Results and Discussion

First, we will look at the contact angle of a droplet on a polymer brush as a function of polymer–liquid affinity for several values of the brush grafting density. Next, we address the pressure anisotropy profiles of brushes and films and examine the influence of the integration limit on the interfacial tensions and resulting Young’s contact angle. We then compare the measured contact angles with the Young’s contact angles. Finally, we explore a route to disentangle the interfacial and bulk contributions to the pressure anisotropy.

### Contact Angles of Droplets on Brushes of Varying Grafting Density

**Figure 3:**
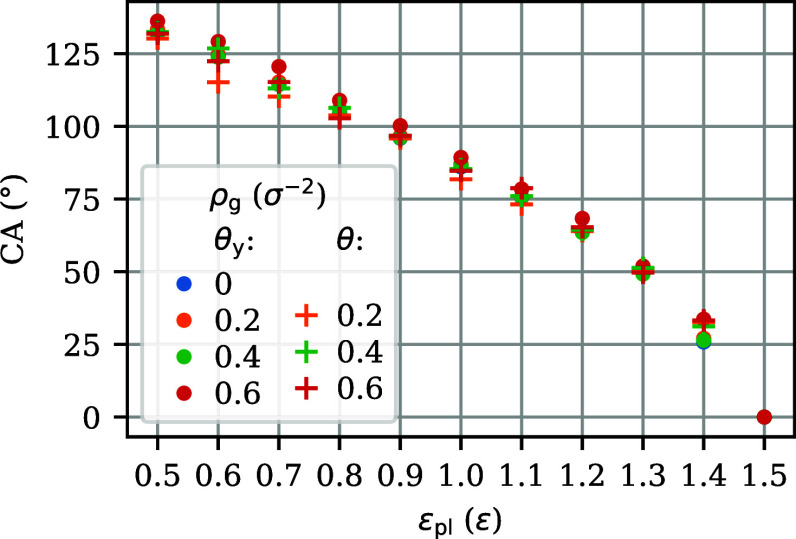
Contact angles as a function of polymer–liquid interaction strength ε_pl_, for polymer brushes at the grafting densities ρ_g_ indicated in the legend and for a nongrafted film denoted by ρ_g_ = 0. Pluses represent contact angles θ measured from simulations of droplets, dots represent contact angles θ_*y*_ calculated from the interfacial tensions using Young’s law. The droplets at ε_pl_ = 1.5 ε are very wide and shallow, rather than the nearly cylindrical shape observed at smaller ε_pl_, which suggests that they are perfectly wetting, θ = 0°. Contact angles for droplets on nongrafted polymer films are not included because they deviate from Young’s law by showing Neumann wetting: the droplet curves the interface underneath and near the droplet.

Now, we can test the validity of eq 6, i.e., the independence of contact angles of droplets from the brush’s grafting density. The contact angles θ measured from the simulations of droplets on brushes over a range of polymer–liquid interaction strengths ε_pl_ are represented in Figure 3 by pluses. The markers at different grafting densities ρ_g_ essentially overlap, indicating that the contact angle of a droplet on the brush is independent of its grafting density for these liquid–brush combinations, in line with previous studies.^34−36^ That is, the difference in surface energies γ_SV_–γ_SL_ is independent of the value of ρ_g_. This implies that the polymer–liquid γ_SL_ and polymer–vacuum γ_SV_ surface energies either are not dependent on the grafting density, in which case there is no Shuttleworth effect, or they have exactly the same grafting density dependence, in which case the Shuttleworth effect is called symmetric.^60^ In view of eq 5, this conveniently implies that in the evaluation of differences between solid–liquid and solid–vapor interfaces, such as in Young’s law, we do not have to distinguish between surface tension Υ and surface free energy density γ, which simplifies the discussion.

### Interfacial Tensions

**Figure 4:**
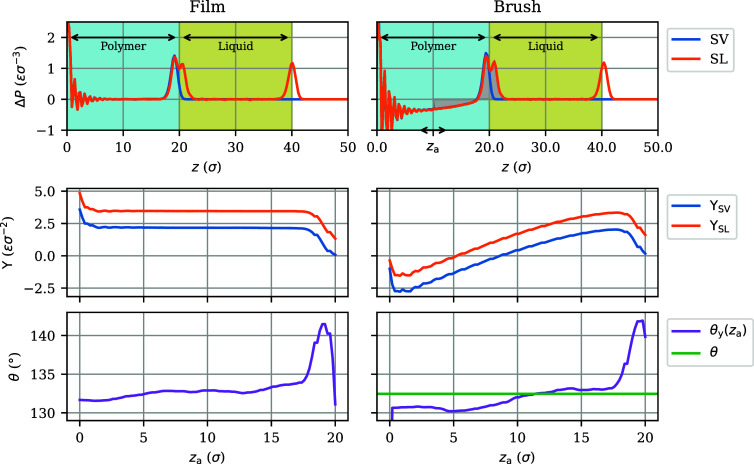
(Top) Pressure anisotropy profiles for (left) an nongrafted film and (right) an chemical identical brush of equal thickness at ρ_g_ = 0.4 σ^–2^, for both polymer–liquid–vapor (SL) and polymer–vapor (SV) stacks, at ε_pl_ = 0.5. The oscillations near *z* = 0 stem from substrate-induced layering. (Center) The corresponding interfacial tensions as functions of the lower integration limit *z*_a_ in eq 2, for *z*_b_ = 30 σ. (Bottom) The resulting Young’s contact angles θ_*y*_, with the horizontal green line denoting the contact angle θ measured for droplets.

The pressure anisotropy of a polymer film and a brush, both with and without a covering fluid layer, is presented in the top row of Figure 4. Since the anisotropy vanishes in the bulk of the liquid layers, the liquid–vapor interfacial tension is readily evaluated as the area under the SL curves, according to eq 2.

The anisotropy also vanishes in the bulk of the polymer film, and consequently, the integral over the polymer–vapor (SV) interface and that over the polymer–liquid (SL) interface are both independent of the lower integration boundary *z*_a_, as long as it lies somewhere in the bulk, as illustrated by wide plateaus in the left-central plot of Figure 4. In the polymer brush, however, grafting induces a negative pressure anisotropy throughout the bulk that steadily becomes more negative with increasing distance from the surface. Consequently, the brush–liquid (SL) and brush–vapor (SV) interfacial tensions calculated using eq 2 depend on the lower integration boundary *z*_a_, as shown in the right-central plot of Figure 4. The plot shows that the values of both Υ_SV_ and Υ_SL_ decrease, and even flip sign, when more of the brush bulk is included in the integral, making it impossible to deduce well-defined values for Υ_SV_ and Υ_SL_.

**Figure 5:**
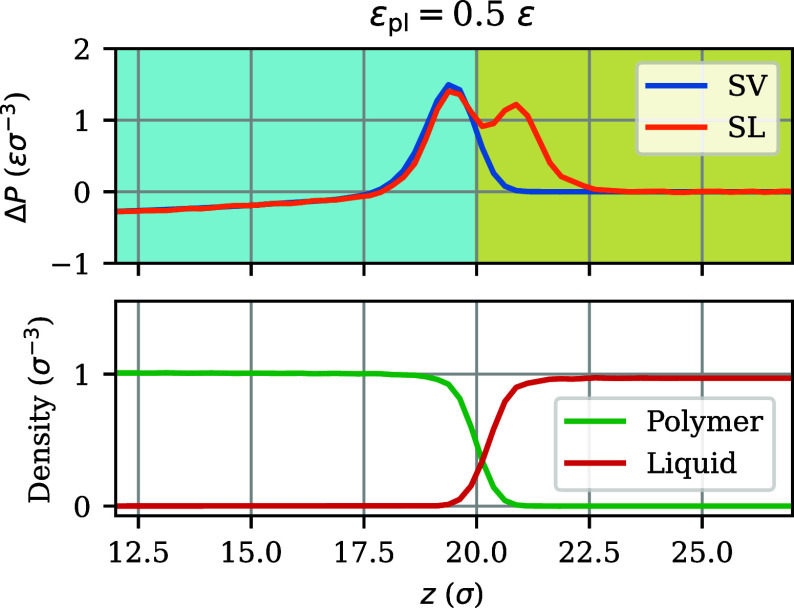
(Top) Zoomed in view of the brush–vapor (SV) and brush–liquid (SL) pressure anisotropy profiles of Figure 4, and (bottom) the density profiles of polymeric and liquid particles for the brush–liquid interface. The pressure anisotropy profiles Δ*P*_SV_ and Δ*P*_SL_ almost overlap in the bulk regions, where the polymer concentration is constant, while they differ in the interfacial regions, where the polymer concentration is not constant.

The difference between the two, however, remains remarkably constant as a consequence of the nearly coalescing Δ*P*_SV_(*z*) and Δ*P*_SL_(*z*) curves in the brush. This point is emphasized by the zoomed-in view of the pressure anisotropy profiles in the interfacial region presented in Figure 5: the two pressure anisotropy curves are almost indistinguishable at distances of more than 2.5 σ from the interface. Hence, the interfacial tension difference Υ_SV_–Υ_SL_ stems solely from the narrow regions surrounding the brush–vapor and brush–liquid interfaces. The largest contribution is seen to occur in the liquid, while the pressure anisotropy in the brush is almost identical in the presence and absence of the liquid. The density distributions in Figure 5 show that the liquid hardly penetrates the brush; the main difference between the pressure anisotropy profiles occurs in the region where the polymer density is not constant.

Returning to the center row of Figure 4, the constant difference between Υ_SV_(*z*_a_) and Υ_SL_(*z*_a_) for the brush over a wide range of *z*_a_ is nearly identical to the constant difference between their counterparts for the polymer film, because the brush and film share similar interfaces. We conclude that, although the brush’s interfacial tensions Υ_SV_ and Υ_SL_ are not well-defined and vary with *z*_a_, their difference is well-defined and takes on a constant value for an integration boundary *z*_a_ sufficiently distanced from the interface.

The Young’s angles θ_*y*_ obtained by inserting the above surface tension differences in eq 6 are plotted in the bottom row of Figure 4. The weak variations of these angles with the integration boundary *z*_a_ are similar in size for brush and film, suggesting that they are probably related to the slow sampling of phase space by polymers. An excellent agreement is observed with the contact angles θ measured from the simulations of droplets. For a collection of films and brushes, the tension differences in the centers of the polymeric layers have been used to calculate the Young’s angles plotted in Figure 3 (dots). The excellent agreement with the contact angles measured from the simulations of droplets validates the use of eq 6, i.e., the assumption that dγ/dρ_g_ is the same for brush–vapor and brush–liquid.

### Interfacial and Bulk Contributions

We have shown above that the interfacial excess of the pressure anisotropy Δ*P* can be identified when considering differences in Υ_SV_ – Υ_SL_. A remaining question, however, is whether we can unambiguously identify the interfacial excess of the pressure anisotropy for a single system, for example, for a given brush–liquid interface. This would enable a definition of the “true” interfacial tension Υ^int^, separating it from the grafting-induced contribution Υ^graft^.

**Figure 6:**
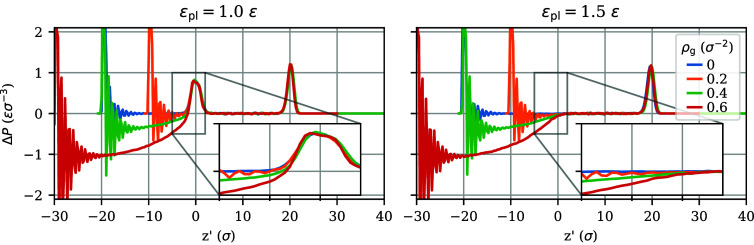
Pressure anisotropy profiles for brushes with several grafting densities, and a nongrafted film, ρ_g_ = 0, in equilibrium with liquid at (left) ε_pl_ = 1.0 ε and (right) ε_pl_ = 1.5 ε. The profiles are presented as functions of the elevation *z*′ = *z* – *H* relative to the polymer–liquid interface, with *H* the height of the polymer layer. The oscillations at the left end of the curves, near *z*′ = −*H*, reflect layering the of the polymer particles near the grafting substrate.

To address this question, Figure 6 reports the pressure anisotropy profiles Δ*P*(*z*) of several brushes with varying grafting densities for a given polymer–liquid interaction strength. The left panel corresponds to ε_pl_ = 1.0 ε, while the right panel corresponds to ε_pl_ = 1.5 ε, equal to the self-interactions. This latter condition makes the tensile peak at the interface vanish, resulting in a purely compressive pressure anisotropy profile. Since the brush thickness varies with the grafting density, the profiles have been shifted with the brush thickness *H*, defined as the inflection point in the polymer density profile, such that their interfacial peaks coincide at *z*′ = *z* – *H* ≈ 0.

The left panel of Figure 6 suggests that the total pressure anisotropy of a brush is a sum of two functions of *z*′, according to
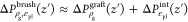
11

In this idealization, to be explored in more detail below, the first term on the right side is a compressive contribution that significantly varies with the grafting density but is independent of the polymer–liquid interaction, while the second is the tensile interfacial contribution that appears to be similar for all ρ_g_ values at a given ε_pl_.

This interfacial contribution is nonzero near the interface only and hence is readily integrated to obtain the interfacial tension Υ^int^. The superposition of grafting and interfacial contributions makes it, in general, impossible to extract Υ^int^ as an integral of Δ*P*^brush^ over a limited range in *z*′. Specifically, the height *z*_0_ where the anisotropy vanishes, Δ*P*^brush^(*z*_0_) = 0, which is sometimes used as an expedient to separate grafting and interfacial contributions, cannot be interpreted as the location where the interface ends.

**Figure 7:**
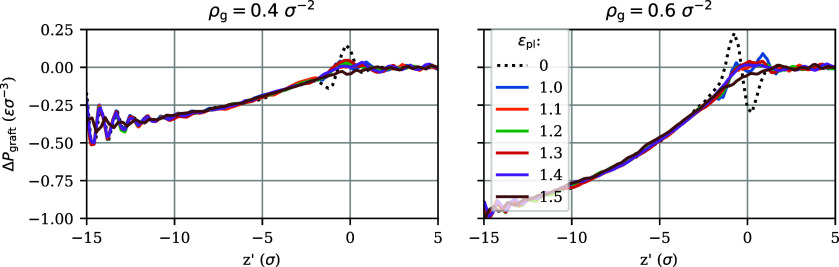
Plots of the pressure anisotropy due to grafting, Δ*P*^graft^(*z*′), for brushes with two grafting densities and various polymer–liquid interaction strengths, calculated as the difference between the pressure anisotropy of the grafted brush and its ungrafted counterpart at the same ε_pl_. Like in Figure 6, the profiles are presented as functions of the elevation *z*′ relative to the polymer–liquid interface. The dashed black line represents the brush–vapor system, which is denoted in the legend by ε_pl_ = 0.

We proceed by exploring the possibility that the interfacial anisotropy is indeed independent of ρ_g_. In this construction, then, by definition, there is no Shuttleworth effect, i.e., dΥ^int^/dρ_g_ = 0. This allows us to explicitly disentangle Δ*P*^int^(*z*′) and Δ*P*^graft^(*z*′). The former can be determined from the nongrafted film, ρ_g_ = 0, and is a function that depends only on ε_pl_. The latter follows from subtraction, Δ*P*^graft^(*z*′) = Δ*P*^brush^(*z*′) – Δ*P*^int^(*z*′). The resulting Δ*P*^graft^(*z*′) are shown in Figure 7, for two values of ρ_g_. The profiles closely overlap for a broad range of ε_pl_. In this range, we thus find a particularly simple scenario: the compressive part of the pressure anisotropy is solely due to grafting, while the tensile part depends only on the interactions near the interface. We note, however, that this scenario runs into its limits for the brush in contact with vapor, see the dashed lines marked ε_pl_ = 0 in Figure 7, for which the obtained Δ*P*^graft^(*z*′) shows a more pronounced oscillation near the interface than its counterparts for the brush–liquid systems. Likewise, a small departure is observed for the limiting case ε_pl_ = 1.5 ε, which is discussed in more detail below. Outside these extreme cases, however, it is possible to disentangle grafting and interfacial contributions by assuming that there is no Shuttleworth effect.

### Autophobic Demixing and Autophobic Dewetting

As shown above, in the specific case where all bead–bead interaction strengths are equal, i.e., ε_pp_ = ε_pl_ = ε_ll_ = 1.5 ε, the peaks in the pressure anisotropy at the polymer–liquid interface vanish (see the right panel of Figure 6). In this case, a nongrafted film mixes with the liquid, since there is no positive interfacial tension. The polymer brush, however, does not mix with the covering liquid layer, even though no tension exists between the two phases. This observation is similar to autophobic demixing, where a liquid layer of polymers does not mix with a brush of identical polymers.^15,54,61−65^ The explanation is that for this athermal system stretching the grafted polymer chains to accommodate solvent molecules in the brush loses more conformational entropy than the entropy to be gained by mixing. It can also be explained in terms of the compressive pressure present in brushes, which prevents any more liquid from entering. Of course, ultimately this argument is equivalent since the pressure in brushes derives from chain elasticity.

Due to the surface tension difference Υ_SV_ – Υ_SL_ being slightly larger than Υ_LV_, spreading is favored and the droplet adopts a very shallow shape with prewetting films on either side. This system provides an interesting situation: there is a phase separation, but it lacks an interfacial tension between the two phases; i.e., no peak of positive Δ*P* is observed in the right panel of Figure 6.

**Figure 8:**
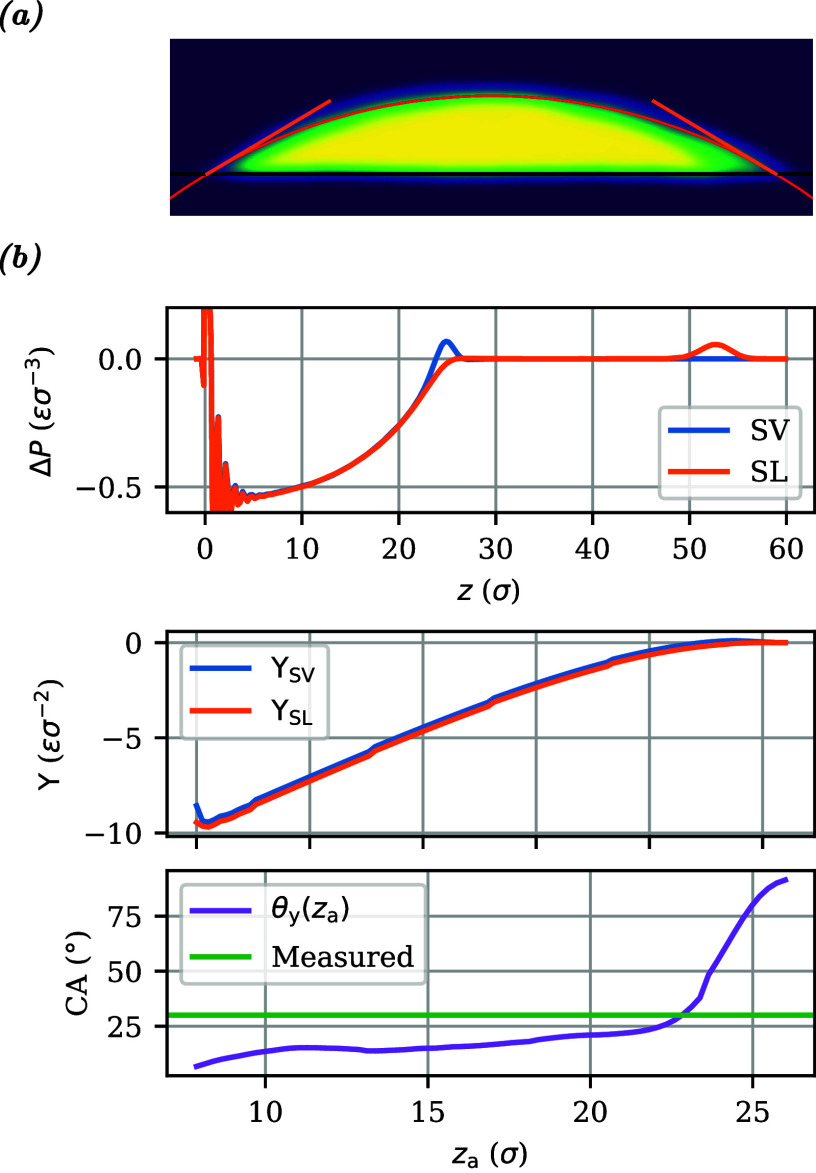
(a) Density profile and circle fit for droplet of 50-mer melt on polymer brush with ρ_g_ = 0.4 σ^–2^ and ε_pp_ = ε_ll_ = ε_pl_ = 0.5 ε. (b) Corresponding pressure anisotropy profiles (top), interfacial tensions as a function of *z*_a_ (middle), and resulting Young’s contact angle (bottom).

Similarly, in autophobic dewetting, a polymer melt only partly wets a brush despite a vanishing interfacial tension.^54,61,64,66,67^ We simulated this phenomenon by using a 50-mer for the wetting liquid, i.e., the same chain length as is used for the brush, with all interaction energies reduced to 0.5 ε to relatively enhance the entropic effects. For this athermal system, a liquid would readily mix when deposited on a nongrafted polymer layer. However, the liquid does not mix with a grafted layer, even though the pressure anisotropy does not show a peak at the interface; see Figure 8. Still, even though the partial wetting is entropic instead of enthalpic in nature here, due to the surface tension difference Υ_SV_ – Υ_SL_ being smaller than Υ_LV_, the liquid forms a droplet. In other words, the partial wetting is a consequence of Υ_LV_ and Υ_SV_, since Υ_SL_ is never positive.

## Conclusions

We investigated the influence of grafting-induced negative pressure anisotropy on the wetting of the polymer brushes. Focusing on systems where the polymer and liquid do not tend to mix, we systematically compared the interfacial tensions determined by integrating the pressure anisotropy profile of planar polymer–liquid interfaces and the difference in interfacial energy probed by extracting contact angles from droplets.

The contact angles of droplets on brushes showed no dependence on the grafting density, implying that if this brush system studied here exhibits a Shuttleworth effect, it must be symmetric. Moreover, they agreed closely with Young’s contact angles computed from interfacial tensions. Investigation of the influence of the lower integration limit in the computation of the interfacial tension showed that the negative contribution to the pressure anisotropy induced by grafting appears equally in Υ_SL_ and Υ_SV_. Furthermore, we showed that it is not possible to divide this system in “bulk” and “interface” at a certain point in *z*, while the grafting-induced pressure exists throughout the bulk, it also extends into the interface. However, we proposed a route to disentangle the interfacial contribution of the pressure from the grafting-induced pressure, which is applicable over a broad range of parameters.

In this work, we have focused on systems with interaction parameters such that little absorption of the wetting liquid into the brush occurs. Future work might extend this investigation to systems in which absorption does occur.

## Data Availability

The data that support the findings of this study are openly available.^68^
